# Angiographic Findings in Patients with Hepatocellular Carcinoma Previously Treated Using Proton Beam Therapy

**DOI:** 10.1155/2019/3580379

**Published:** 2019-07-04

**Authors:** Hiroaki Takahashi, Kensaku Mori, Yuta Sekino, Toshiyuki Okumura, Takashi Hiyama, Kuniaki Fukuda, Naoyuki Hasegawa, Masafumi Sakai, Shunsuke Kikuchi, Yohei Takei, Takashi Iizumi, Hideyuki Sakurai, Manabu Minami

**Affiliations:** ^1^University of Tsukuba Hospital, Department of Diagnostic and Interventional Radiology, Ibaraki, Japan; ^2^Ibaraki Prefectural Central Hospital, Department of Diagnostic Radiology, Ibaraki, Japan; ^3^University of Tsukuba Hospital, Department of Radiation Oncology and Proton Medical Research Center, Ibaraki, Japan; ^4^National Cancer Center Hospital East, Department of Diagnostic Radiology, Chiba, Japan; ^5^University of Tsukuba Hospital, Department of Gastroenterology, Ibaraki, Japan

## Abstract

Given the growing interest in using proton beam therapy (PBT) for hepatocellular carcinoma (HCC), it is possible that transarterial chemoembolization (TACE) could be used for selected patients who have previously undergone PBT. However, these cases can be technically challenging to treat and require appropriate preparation. Thus, we aimed to identify angiographic findings in this setting. We retrospectively identified 31 patients (28 men and 3 women, mean age: 69 years, range: 43–84 years) who underwent hepatic angiography plus TACE or transarterial infusion chemotherapy (TAI) for HCC that recurred after PBT (July 2007 to June 2018). We discovered four angiographic findings, which we speculate were related to the previous PBT. 18 patients experienced recurrence in the irradiated field, and 13 patients experienced recurrence outside the irradiated field. 29 patients underwent TACE and only 2 patients underwent TAI. The mean number of previous PBT treatments was 1.3 ± 0.6 (range: 1–4). The median interval from the earliest PBT treatment to hepatic angiography was 559 days (range: 34–5,383 days), and the median interval from the latest PBT treatment to hepatic angiography was 464 days (range: 34–5,383 days). Abnormal staining of the irradiated liver parenchyma was observed in 22 patients, which obscured the angiographic tumor staining in 4 patients. Development of a tortuous tumor feeder vessel was observed in 13 patients. Development of an extrahepatic collateral pathway was observed in 7 patients. Development of an arterioportal or arteriovenous shunt was observed in 4 patients. Based on these findings, we conclude that PBT was associated with various angiographic findings during subsequent transarterial chemotherapy for recurrent HCC, and familiarity with these findings will be important in developing appropriate treatment plans.

## 1. Introduction

Hepatocellular carcinoma (HCC) is the most common primary cancer of the liver [1]. According to the NCCN guidelines, there are numerous strategies for treating HCC, including resection, transplantation, radiofrequency ablation, transarterial chemoembolization (TACE), radiotherapy (RT), and systemic therapy using sorafenib or lenvatinib [2]. All patients with HCC should be evaluated for potential curative therapies, including resection and transplantation [2]. Locoregional therapy, including ablation, TACE, and RT, is indicated for patients who are not candidates for curative therapy or indicated as a bridge therapy for patients who are candidates for transplantation [2].

Recent reports have described favorable clinical outcomes after proton beam therapy (PBT) for HCC, based on a 5-year overall survival rate of 24–48% [3, 4] and a 5-year local control rate of approximately 80% [3, 5]. Among the external beam RT modalities, PBT may be superior to X-ray therapy based on excellent dose localization to the therapeutic target [1]. Furthermore, given the good outcomes after PBT for HCC, some patients may be eligible for TACE treatment of intrahepatic HCC metastasis, while repeated PBT, TACE, or systemic therapy may be feasible in cases of local recurrence after PBT. Moreover, PBT is effective for patients with HCC who also have portal vein tumor thrombus (PVTT) [6], and the efficacy of combined therapy of TACE and RT for HCC with PVTT has been reported [7, 8]. Therefore, given the growing interest in using PBT for HCC, it is possible that TACE could be used for selected patients who have previously undergone PBT.

The purpose of our study is to evaluate the angiographic findings from patients with HCC previously treated using PBT. We classified abnormal PBT-related angiographic findings and analyzed these factors' frequency, onset timing, and influence on technical difficulty.

## 2. Materials and Methods

This study's retrospective protocol was approved by our institutional review board. The requirement for informed consent was waived.

### 2.1. Patient Acquisition

We identified patients who underwent transarterial chemotherapy for HCC that recurred after PBT between July 2007 and June 2018. 37 patients fulfilled the inclusion criteria: (1) diagnosis of HCC was confirmed pathologically or clinically according to the accepted guidelines [9], (2) PBT was performed for HCC before the transarterial chemotherapy, and (3) TACE or transarterial infusion chemotherapy (TAI) was performed. However, 6 patients were excluded because hepatic resection (n=3) or radiofrequency ablation (n=3) had been performed before the transarterial chemotherapy. Thus, the present study included 31 patients (28 men and 3 women, mean age: 69 years, range: 43–84 years) who underwent TACE or TAI after PBT. The patients' records were reviewed to determine the type of transarterial chemotherapy, the number of previous PBT treatments, and the interval between the angiography and the previous PBT treatment(s).

### 2.2. Hepatic Angiography and Transarterial Chemotherapy

All patients routinely underwent digital subtraction angiography of the celiac trunk and/or superior mesenteric artery, as well as cone-beam computed tomography (CBCT) of the proper or common hepatic artery. Angiography of an extrahepatic artery was performed if the HCC was not observed using hepatic arteriography or CBCT. The TACE and TAI treatments involve a mixture of 4–10 mL of iodized oil (Lipiodol; Andre Guerbet, Aulnay-sous-Bois, France) and the chemotherapeutic agent (cisplatin up to 50 mg or epirubicin up to 50 mg and mitomycin C up to 10 mg). Gelatin sponge particles (Gelpart; Nihonkayaku, Japan) were also used during the TACE treatment. Both procedures were performed using 3–4-Fr shepherd hook catheters and microcatheters with a tip diameter of 1.7–1.9 Fr.

### 2.3. PBT Procedure

Treatment planning for PBT was performed as previously reported [3, 10]. The irradiation protocols were generally classified according to tumor location: (1) a total dose of 77.0 GyE in 35 fractions for tumors located <2 cm from a gastrointestinal organ, (2) a total dose of 72.6 GyE in 22 fractions for tumors located <2 cm from the porta hepatis, or (3) a total dose of 66.0 GyE in 10 fractions for peripheral tumors >2 cm from both the GI tract and porta hepatis. The protocols were adjusted in some cases to avoid excessive irradiation of the adjacent organs. All patients received PBT on 5 days per week.

### 2.4. Image Analysis

We analyzed the PBT-related angiographic findings in each case (based on their relationship to the PBT irradiation field) and categorized them into four types: (1) abnormal staining of the irradiated liver parenchyma, (2) development of tortuous tumor feeder vessels, (3) development of an extrahepatic collateral pathway to the liver, and (4) development of arterioportal (AP) or arteriovenous (AV) shunts. Intrahepatic and extrahepatic shunts observed in the PBT irradiation field were both included in the study. The four PBT-related angiographic findings were counted for each case, although findings that were not clearly related to the PBT irradiation field were not considered. The HCCs targeted during TACE or TAI were classified according to whether they were in or outside the previous PBT irradiation field. All characteristics were judged based on mutual agreement between two radiologists (HT: 8 years of experience, KM: 25 years of experience).

### 2.5. Statistical Analysis

Differences in the distributions of the angiographic findings were evaluated using the chi-squared test. All data were analyzed using R software (version 3.3.2). Differences were considered statistically significant at P-values of <0.05.

## 3. Results

Table 1 shows the 31 patients' characteristics. 18 patients experienced recurrence in the irradiated field and 13 patients experienced recurrence outside the irradiated field. Most patients underwent TACE (29 patients) and only 2 patients underwent TAI. The average age was 68.5 ± 9.8 years (range: 43–84 years). The mean number of previous PBT treatments was 1.3 ± 0.6 (range: 1–4). The median interval from the earliest PBT treatment to the angiography was 559 days (range: 34–5,383 days), and the median interval from the latest PBT treatment was 464 days (range: 34–5,383 days).

**Figure 1:**
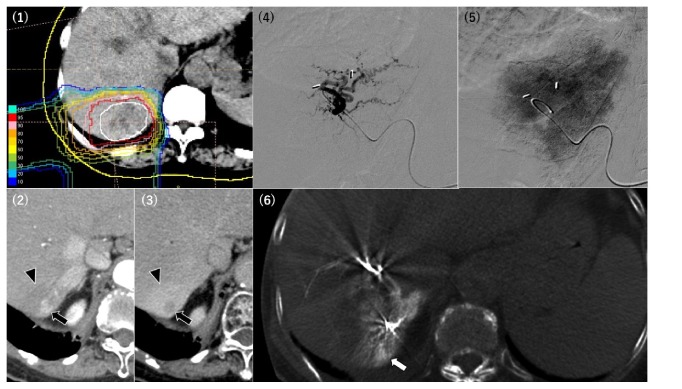
A 70-year-old woman underwent her first and second proton beam therapy (PBT) treatment for hepatocellular carcinoma (HCC) in S7 43 months (1) and 16 months (not shown) before the angiography, respectively. Dynamic computed tomography before the transarterial chemoembolization revealed local recurrence of the PBT-treated HCC, with early enhancement at the hepatic arterial phase and washout at the equilibrium phase (arrow). The surrounding irradiated parenchyma exhibited delayed enhancement (arrowhead) (2,3). A tortuous tumor feeder was noted during the procedure (4), although enhancement of the recurrent HCC was obscured by the abnormal staining of the irradiated liver parenchyma (5). Cone-beam computed tomography clearly showed that the selected artery fed the recurrent tumor (white arrow) (6).

**Figure 2:**
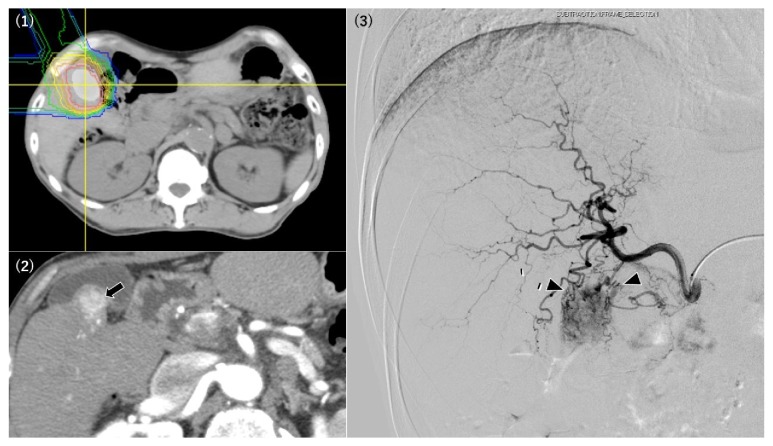
A 55-year-old man underwent proton beam therapy (PBT) for hepatocellular carcinoma (HCC) in S5 29 months before the angiography procedure (1). Dynamic computed tomography revealed local recurrence of the PBT-treated HCC (arrow) at the hepatic arterial phase (2). The recurrent HCC exhibited clear tumor staining during the procedure, with several tortuous vessels feeding the lesion (arrowhead), which made it technically difficult to perform selective catheterization (3).

**Figure 3:**
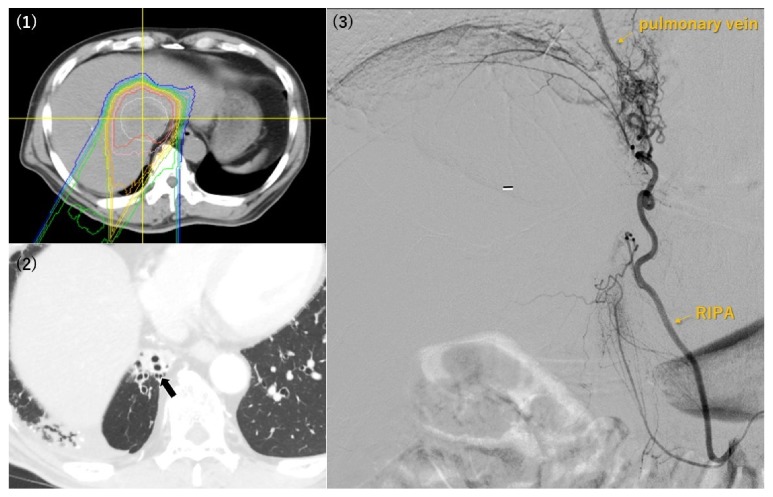
A 66-year-old man underwent his first proton beam therapy (PBT) treatment for hepatocellular carcinoma (HCC) in S8/1 48 months before the angiography procedure (1). Computed tomography revealed radiation-induced pneumonitis in the medial side of the right lower lung (2). An extrahepatic collateral pathway (via right inferior phrenic artery, RIPA) and an arteriovenous shunt for the pulmonary vein were noted during the procedure (3). The location of the arteriovenous shunt corresponded to the site of the radiation-induced pneumonitis.

The relationships between the PBT-related angiographic findings and the HCC characteristics are summarized in Table 2. In the PBT irradiation fields, we identified four angiographic findings: (1) abnormal staining of the irradiated liver parenchyma, (2) development of a tortuous tumor feeder vessel, (3) development of an extrahepatic collateral pathway, and (4) development of AP or AV shunts. Abnormal staining of the irradiated liver parenchyma was observed in 22 patients, and the angiographic tumor staining was obscured by the abnormal parenchymal staining in 4 patients (Figure 1). In all 4 patients, the CBCT could identify the tumors and their feeding arteries. Among the 22 patients with abnormal parenchymal staining, the median interval from the earliest PBT treatment to the angiography was 629 days (range: 109–3,163 days) and the median time from the latest PBT treatment was 466.5 days (range: 109–3,163 days) (Table 3). Development of a tortuous tumor feeder vessel was observed in 13 patients, and in all cases the HCCs had recurred in the PBT irradiation field (Figures 1 and 2, Table 2). Among these 13 patients, the median interval from the earliest PBT treatment to the angiography was 911 days (range: 381–2,938 days), and the median interval from the latest PBT treatment was 559 days (range: 381–2,938 days) (Table 3). Development of an extrahepatic collateral pathway was observed in 7 patients, which involved the right inferior phrenic artery (6 patients) (Figure 3) or the omental artery (1 patient), and all collateral pathways fed the irradiated HCC or/and irradiated liver parenchyma. All the 7 patients only underwent a single PBT treatment, and the median intervals from the PBT treatments to the angiography were both 917 days (range: 418–3,163 days) (Table 3). Development of AP or AV shunts was observed in 4 patients, which involved an AP shunt (2 patients), an AV shunt to the hepatic vein (1 patient), and an AV shunt to the pulmonary vein (1 patient) (Figure 3). All the 4 patients only underwent a single PBT treatment, and the median intervals from the earliest and latest PBT treatments to the angiography were both 588 days (range: 397–1,443 days) (Table 3).

## 4. Discussion

The present study identified four angiographic findings that were observed during transarterial chemotherapy, which was performed for HCCs that recurred after PBT. Interestingly, these four findings seem to appear at different intervals after the PBT. Among patients who underwent a single PBT treatment, the earliest finding was abnormal staining of the liver parenchyma (median interval: 477 days), the development of AV or AP shunts occurred later (median interval: 588 days), the development of tortuous feeder vessels occurred even later (median interval: 699 days), and the development of extrahepatic collateral pathways was the latest finding (median interval: 917 days).

Abnormal staining of irradiated liver parenchyma was the most common PBT-related angiographic finding in our study (22 out of 31 cases, 71%), which appeared as an area with dense and prolonged staining. Irradiated hepatic parenchyma after PBT can be observed as low-attenuation areas on noncontrast CT or areas with early and prolonged enhancement on dynamic CT [11]. During angiography-assisted CT, irradiated parenchyma exhibits decreased attenuation on CT arterial portography and increased attenuation on CT arteriography, which is the result of an arterial-predominant blood supply to the irradiated parenchyma (caused by radiation-induced venoocclusive disease) [11–13]. Prolonged enhancement of the irradiated parenchyma is related to contrast agent retention in the fibrous tissue [14]. Furthermore, a previous study demonstrated that the earliest disappearance of radiation-induced hepatic injury on imaging was observed 42 months after the PBT [11–13], which suggests that the irradiated parenchyma might have diminished arterial supply that persists long after the PBT treatment. These previously reported results agree with our findings, as we found early and prolonged abnormal staining of the irradiated parenchyma during angiography, which occurred at a relatively short interval after PBT. Moreover, we found that the irradiated liver parenchyma appeared as a pseudo-lesion in some cases and obscured the tumor staining in other cases. When the abnormal parenchymal staining obscured the tumor staining, CBCT was useful for detecting the tumor and its feeder vessels [15, 16].

Development of tortuous tumor feeder vessels was another PBT-related angiographic finding in the present study, and this characteristic was exclusively observed when the TACE or TAI targeted HCCs in the PBT irradiated field. Therefore, development of tortuous tumor feeder is likely to be associated with PBT-treated HCCs local recurrence. In addition, a previous report has described the local control rates of PBT-treated HCC (1-year: 98%, 3-year: 87%, 5-year: 81%) [3], which seems to be aligned with the mid-to-late development of tortuous tumor feeder vessels. According to our clinical experience, we speculate that selective catheterization becomes technically difficult when these vessels are present. Therefore, careful planning and attention are necessary when treating HCCs in the PBT irradiated field.

The present study revealed that extrahepatic collateral development could occur after PBT. In this context, previous reports have indicated that repeated transcatheter intervention could cause hepatic artery occlusion and the development of extrahepatic collaterals [17–19]. In our study, the right inferior phrenic artery or omental artery was developed to supply the irradiated liver parenchyma, without obstruction of any hepatic arteries. Thus, it is possible that extrahepatic collateral development after PBT might be the result of diaphragm or omentum irradiation. Furthermore, embolization of the adjacent organs should be avoided for patients who require embolization through an extrahepatic collateral feeder vessel [17]. Therefore, it is important to identify any extrahepatic collateral arteries using the pretreatment CT when planning TACE or TAI for patients with HCC that were previously treated using PBT.

Attention should also be paid to the development of AP and AV shunts, although it was less frequent than the other findings in patients with PBT-treated HCC. Nevertheless, TACE for HCC with AP or AV shunts can increase the risk of life-threatening complications, such as lung damage and pulmonary embolism [18, 20]. Lipiodol-related cerebral embolism is also known to occur in cases with artery-pulmonary vein shunts [21]. Thus, efflux of the embolic agent into the shunts should be avoided [20, 22], which might be achieved via prophylactic embolization of the AP shunt before TACE [22, 23]. Balloon-assisted TACE might also be useful when treating cases with AP shunts [22, 24].

The present study has several limitations. First, we did not evaluate angiographic findings before PBT, which raises the possibility that some of the angiographic findings were present before PBT (especially extrahepatic collateral pathways or AP and AV shunts). Second, the intervals between PBT and transarterial chemotherapy varied in each case. Thus, in order to accurately assess the onset period for each finding, it would be preferable to perform angiography at defined intervals after PBT. Third, the relationship between the radiation dose and angiographic findings could not be analyzed, as some of patients underwent multiple PBT treatments. Thus, well-designed prospective studies are needed to address these limitations and validate our findings.

## 5. Conclusions

In conclusion, PBT was associated with various angiographic findings that were observed during transarterial chemotherapy for HCC recurrence. Irradiated liver parenchyma appeared as a pseudo-lesion during angiography or obscured the tumor staining in some cases. In addition, tortuous tumor feeder vessels, extrahepatic collateral pathways, and AV and AP shunts were also observed. Familiarity with these angiographic findings may help radiologists develop appropriate treatment plans for patients with PBT-treated HCC recurrence.

## Figures and Tables

**Table 1:** Patient characteristics.

*Total patients*	31
*Procedure*	
TACE	29 (93.5)
TAI	2 (6.5)
*Male sex*	28 (90.3)
*Age at TACE or TAI, years*	68.5 ± 9.8 (43–84)
*Child-Pugh*	
A	28 (90.3)
B	3 (9.7)
C	0 (0)
*Previous PBT treatments*	
1	25 (80.6)
2	5 (16.1)
3	0 (0)
4	1 (3.2)
*Diameter of HCC treated using PBT, mm*	37.5 ± 26.6 (8-122)
*Median follow-up time after earliest PBT, days*	559 (34–5,383)
*Median follow-up time after latest PBT, days*	464 (34–5,383)
*Targeted HCCs in TACE or TAI*	
Irradiated HCCs targeted	18
Irradiated HCCs not targeted	13
*PBT-related findings*	
Abnormal staining of irradiated liver parenchyma	22 (71.0)
Development of tortuous tumor feeder	13 (41.9)
Development of extrahepatic collateral pathway	7 (22.6)
Development of AP/AV shunt	4 (12.9)

Data are reported as number (percentage), median (range) or mean ± standard deviation.

TACE: transarterial chemoembolization, TAI: transcatheter arterial infusion, PBT: proton beam therapy, HCC: hepatocellular carcinoma, AP/AV: arterio-portal or arteriovenous.

**Table 2:** The prevalence of PBT-related angiographic findings at the initial hepatic angiography according to HCC targeting.

Angiographic findings	Total (n=31)	Irradiated HCCs targeted during TACE or TAI (n=18)	Irradiated HCCs NOT targeted during TACE or TAI (n=13)	*P*
Abnormal staining of irradiated liver parenchyma	22 (71%)	14 (78%)	8 (62%)	0.326
Development of tortuous tumor feeder	13 (42%)	13 (72%)	0 (0%)	<0.001
Development of extrahepatic collateral pathway	7 (23%)	5 (28%)	2 (15%)	0.415
Development of AP/AV shunt	4 (13%)	4 (22%)	0 (0%)	0.069

The chi-squared test was used to calculate p-values. Irradiated HCCs refer to the TACE/TAI-targeted tumors being within the PBT irradiation field.

TACE: transarterial chemoembolization, TAI: transcatheter arterial infusion, PBT: proton beam therapy, HCC: hepatocellular carcinoma, AP/AV: arterio-portal or arteriovenous.

**Table 3:** Relationship between PBT-related angiographic findings and the elapsed time after PBT treatment.

	All patients		Single PBT treatment (n=25)		Multiple PBT treatments (n=6)		
	Follow-up after first PBT, days	Follow-up after last PBT, days	N	Follow-up after PBT, days	N	Follow-up after first PBT, days	Follow-up after last PBT, days
Abnormal staining of irradiated liver parenchyma (n=22)	629 (109–3,163)	466.5 (109–3,163)	17	477 (109–3,163)	5	225 (464–720)	877 (1,274–1,950)

Development of tortuous tumor feeder (n=13)	911 (381–2,938)	559 (381–2,938)	9	699 (381–2,938)	4	466.5 (441–720)	1,284.5 (911–1,950)

Development of extrahepatic collateral pathway (n=7)	917 (418–31,63)	917 (418–3,163)	7	917 (418–3,163)	0	N/A	N/A

Development of AP/AV shunt (n=4)	588 (397–1,443)	588 (397–1,443)	4	588 (397–1,443)	0	N/A	N/A

Data are reported as median (range).

TACE: transarterial chemoembolization, TAI: transcatheter arterial infusion, PBT: proton beam therapy, HCC: hepatocellular carcinoma, AP/AV: arterio-portal or arteriovenous.

## Data Availability

The data used to support the findings of this study are available from the corresponding author upon request.

## Abbreviations

RT: Radiotherapy
PBT: Proton beam therapy
TACE: Transarterial chemoembolization
TAI: Transarterial infusion chemotherapy
AP: Arterioportal
AV: Arteriovenous
CBCT: Cone-beam CT
HCC: Hepatocellular carcinoma.
